# Facial emotion recognition in patients with depression compared to healthy controls when using human avatars

**DOI:** 10.1038/s41598-023-31277-5

**Published:** 2023-04-12

**Authors:** Marta Monferrer, Arturo S. García, Jorge J. Ricarte, María J. Montes, Antonio Fernández-Caballero, Patricia Fernández-Sotos

**Affiliations:** 1 Servicio de Salud de Castilla-La Mancha, Complejo Hospitalario Universitario de Albacete, Servicio de Salud Mental, 02004 Albacete, Spain; 2 Departmento de Sistemas Informáticos, Universidad de Castilla-La Mancha, 02071 Albacete, Spain; 3 Neurocognition and Emotion Unit, Instituto de Investigación en Informática de Albacete, 02071 Albacete, Spain; 4 Departmento de Psicología, Universidad de Castilla-La Mancha, 02071 Albacete, Spain; 5 CIBERSAM-ISCIII (Biomedical Research Networking Centre in Mental Health, Instituto de Salud Carlos III), 28016 Madrid, Spain

**Keywords:** Psychology, Engineering

## Abstract

The negative, mood-congruent cognitive bias described in depression, as well as excessive rumination, have been found to interfere with emotional processing. This study focuses on the assessment of facial recognition of emotions in patients with depression through a new set of dynamic virtual faces (DVFs). The sample consisted of 54 stable patients compared to 54 healthy controls. The experiment consisted in an emotion recognition task using non-immersive virtual reality (VR) with DVFs of six basic emotions and neutral expression. Patients with depression showed a worst performance in facial affect recognition compared to healthy controls. Age of onset was negatively correlated with emotion recognition and no correlation was observed for duration of illness or number of lifetime hospitalizations. There was no correlation for the depression group between emotion recognition and degree of psychopathology, excessive rumination, degree of functioning, or quality of life. Hence, it is important to improve and validate VR tools for emotion recognition to achieve greater methodological homogeneity of studies and to be able to establish more conclusive results.

## Introduction

Depressive disorders are one of the most prevalent causes of disability globally^1^, impacting over 300 million individuals with a prevalence in the general population of around 20% at least once in a lifetime^2^. The Diagnostic and Statistical Manual of Mental Disorders (DSM-5) defines depressive disorders as a diagnostic category characterized by common features such as affective, cognitive, and neurovegetative changes, with the primary symptoms being a sad, empty, or irritable mood and somatic and cognitive alterations that significantly affect an individual’s functioning^3^. Beck’s cognitive theory^4^, which emphasizes the role of an individual’s thoughts, interpretations, and attitudes in the pathogenesis of depression, is the most commonly accepted explanatory model of depression. This theory posits that the cognitive triad of depression, which includes a negative view of the self, the future, and the world, underlies the disorder^5^.

Social cognition, which encompasses the mental operations involved in identifying, perceiving, and interpreting social information and facilitating interpersonal interaction, is composed of four domains: emotional processing, theory of mind, social perception, and attributional style^6^. It is precisely the negative, mood-congruent, and therefore cognitively biased thinking found in depression that appears to interfere with social cognition^7^, particularly with attributional style but also emotional processing and theory of mind^8–11^. These deficits in social cognition can lead to isolation, which is a risk factor for the onset and maintenance of the illness^12^, as well as its long-term severity, since it clearly interferes with social support (perceived and real), which, if low, can become a high risk factor for developing a first major depressive episode and its subsequent recurrence^13,14^.

This article focuses on emotional processing, which is defined as the ability to identify, facilitate, regulate, understand, and manage emotions^15^. Emotional processing is divided into three subdomains: emotional understanding and emotional management, which are classified as higher perceptual level processes, and emotional facial recognition, which is considered a lower level process. Emotional facial recognition, or the ability to identify and categorize emotional states through facial expressions and non-facial signals such as the voice^16^, is considered the initial and most basic step in the whole process involving social cognition. In depression, there is a negative and mood-congruent cognitive bias that manifests as a preference for negative emotional information in memory encoding and recall tasks, slower reaction times when naming negative emotional words in the emotional Stroop task or responding to happy targets in the emotional Go/No-Go task, and a tendency to perceive and categorize ambiguous facial expressions as “negative emotions” in tests such as the Facial Emotions Recognition Test (FERT).

Additionally, certain cognitive styles, such as excessive rumination, have been associated with depression^17^. Excessive rumination is characterized as a type of maladaptive cognitive processing with depressive autofocus, a tendency to repetitive thinking about one’s own depressive symptoms and interpersonal problems, their possible causes, meanings, and implications^18^. This has been linked to deficits in social cognition^19^ and even in healthy people it has been related to a negative cognitive bias and negative evaluation of faces^20^. Research on social cognition in depression has primarily focused on studying facial emotion recognition, finding an inverse association with the level of psychopathological severity of depression. This association has been found in both the decompensation and euthymia phases^21,22^. Traditionally, a deficit in the ability to recognize facial emotions has been commonly observed in individuals with depression. This deficit is typically characterized by an ability to correctly identify sadness, but difficulty in identifying neutral and ambiguous expressions, which are often perceived as sadder or less happy. These observations are frequently attributed to the negative cognitive bias present in depression, and have been observed in both behavioral data^23^ and neural activity during emotion recognition tasks^24^.

The increased interest in social cognition, particularly facial emotion recognition, has prompted researchers to develop various evaluation tools and methods of classification. These include three main approaches: (1) non-behavior-dependent methodologies such as MRI and electroencephalography, (2) behavioral instruments, and (3) instruments or methods used to assess facial recognition impairment in health conditions^25^. Behavioral instruments have been the most widely used and developed. Initially, most behavioral tools were created in a natural format, using images or photographs. Later, videos were also used to present more realistic and genuine facial expressions, in an attempt to increase the validity of static stimuli^26^. However, while some videos have become standardized measures, most have not been validated and present various limitations in terms of format^27^. In recent years, behavioral instruments have focused on virtual reality (VR), which allows for the creation of controlled and practically real environments and situations^28–31^. This has led to the development of new computerized interventions and assessment tools for mental disorders with increased accessibility and ecological validity, making VR an attractive tool for clinical practice^32–34^.

Our multidisciplinary research team is working on developing a psychological intervention using VR to improve social cognition in individuals with major depressive disorder (MDD). However, for this work, it was decided to use a non-immersive desktop VR application due to concerns that patients would experience an overly uncomfortable moment and/or mild adverse events (e.g., headache, giddiness, and VR misuse behavior), as described in previous studies^35,36^. Although immersion is one of the most important aspects of VR technology, less immersive VR such as desktop VR has also received considerable attention^37–39^, especially in cases where higher levels of immersion are not recommended or possible. Currently, we are working on therapies with virtual humans that will involve the use of immersive VR equipment. However, this will be done in a step-wise manner. This means that patients will start by facing avatars on a computer screen, while increasing the level of immersion over time using head-mounted displays.

To achieve the aforementioned goal, several logical methodological steps have been taken. Firstly, the six basic emotions were represented by a new set of dynamic virtual faces (DVFs) designed using the action units of the FACS system^26^. Secondly, these faces were shown to 204 healthy individuals, and it was found that the DVFs were valid for accurately recreating facial expressions of human emotions^40^. In line of this, the current study focuses on examining facial emotion recognition scores through DVFs in 54 stable patients diagnosed with MDD, and comparing these scores to a group of healthy controls from the same demographic area. This is done in order to identify specific difficulties and develop more effective targeted interventions. The following hypotheses have been established:

- Hypothesis 1 (H1): Patients with MDD will demonstrate a lower rate of emotional recognition compared to healthy controls.
- Hypothesis 2 (H2): The MDD group will demonstrate a higher rate of recognition for sadness compared to the other emotions.
- Hypothesis 3 (H3): For the MDD group, no association will be observed between emotional recognition and age of onset, duration of illness, and number of hospitalizations throughout life.
- Hypothesis 4 (H4): For the MDD group, an association will be observed between emotional recognition and degree of psychopathology, excessive rumination, degree of functioning, and quality of life.

## Results

### Comparison of recognition scores between depression and healthy groups in emotion recognition (H1)

Regarding recognition scores, differences were found among both groups. The depression group presented a lower rate of emotional recognition than the healthy group, shown by the analyses carried out through the Mann–Whitney U test ($U=$ 555.0, $p<$ 0.001). The results of the recognition scores for healthy controls and patients is summarized in Table 1.

**Table 1:** Emotion recognition scores for each emotion depicted for the depression group and healthy controls.

MDD group	Neutral (%)	Surprise (%)	Fear (%)	Anger (%)	Disgust (%)	Joy (%)	Sadness (%)
Neutral	90.3	2.8	0.9	1.4	0.5	0.5	3.7
Surprise	2.3	89.6	4.4	1.4	0.2	0.9	1.2
Fear	1.2	41.7	48.4	2.8	1.9	0.2	3.9
Anger	2.1	5.1	2.5	83.6	5.3	0.0	1.4
Disgust	1.2	7.2	4.2	19.9	66.9	0.2	0.5
Joy	6.0	4.2	0.9	1.4	2.3	84.5	0.7
Sadness	7.9	7.6	8.1	8.1	4.4	0.9	63.0

Healthy group	Neutral (%)	Surprise (%)	Fear (%)	Anger (%)	Disgust (%)	Joy (%)	Sadness(%)
Neutral	94.0	0.5	0.5	0.0	0.9	0.0	4.2
Surprise	0.9	90.3	8.3	0.0	0.0	0.0	0.5
Fear	0.9	12.7	77.3	0.2	0.7	0.0	8.1
Anger	0.7	1.2	1.9	92.4	3.0	0.0	0.9
Disgust	0.2	0.5	0.9	13.2	85.0	0.0	0.2
Joy	4.9	0.7	0.2	0.7	0.5	93.1	0.0
Sadness	3.0	3.5	5.6	0.9	1.6	0.0	85.4

Columns: recognized emotions.

Rows: displayed emotions.

The average score for healthy controls is 88.19%, while it is 75.17% for the depression group. The greatest differences were in fear, with a score of 48.4% for the depression group and 77.3% for healthy controls; sadness, 63.0% and 83.4%; and disgust, 66.9% and 85.0%. In all cases, the score is higher for the control group. The differences in hits between the two groups cannot be attributed to a bias in the recognition of the 52 DVFs. It was not found that the number of recognition errors was very high for some DVFs or that there were obvious recognition differences between the two groups for certain specific DVFs.

### Recognition scores for depressed and healthy groups in relation to age, gender and education level

The Kruskal–Wallis test revealed significant differences ($\chi ^2_{(2)}$ = 15.487, $p<$ 0.001) among the three age groups, consisting of young ($n=$ 9), middle-aged ($n=$ 27) and elderly ($n=$ 18), for the hits in the depression group. There was a lower recognition score for the elderly compared to the young ($p=$ 0.002) and middle-aged ($p=$ 0.004). Statistically significant results were also found using Spearman’s rank correlation coefficient ($r= -$ 0.628, $p<$ 0.001) as a negative correlation was found indicating that the older the participant the fewer hits. As for the healthy group with the same three categories, young ($n=$ 10), middle-aged ($n=$ 27) and elderly ($n=$ 17), no significant differences were found ($\chi ^2_{(2)}$ = 0.135, $p=$ 0.935). In this case, the Spearman test showed a similar but not significant negative correlation ($r=-$ 0.050, $p=$ 0.722) when age augmented.

Regarding gender (34 females, 20 males), no statistically significant differences were found using the Mann–Whitney U test, neither for the MDD group ($U=$ 311.5, $p=$ 0.609) nor for the healthy group ($U=$ 367.5, $p=$ 0.620). For education level based on the categories basic ($n=$ 17), medium ($n=$ 21) and high ($n=$ 16), no statistically significant differences were found for the depression group using the Kruskal–Wallis test ($\chi ^2_{(2)}$ = 3.274, $p=$ 0.195). However, significant differences were found for the healthy group ($\chi ^2_{(2)}$ = 10.923, $p=$ 0.004). There are more hits for medium education level than for low ($p=$ 0.09) and high ($p=$ 0.026).

### Recognition scores for the depression group (H2)

Recognition scores for the individual emotions in the depression group were analyzed by the Friedman test, and significant differences were found ($\chi ^2_{(6)}$ = 167.671, $p<$ 0.001). Specifically, results for the emotional recognition of sadness were also compared to the rest of basic emotions, and some significant differences were found, described to be worse recognized than neutral ($p<$ 0.001), anger ($p<$ 0.001), joy ($p<$ 0.001) and surprise ($p<$ 0.001). No significant differences were obtained between sadness and fear ($p=$ 0.249), and sadness and disgust ($p=$ 1.000).

### Influence of age of onset, duration of illness and the number of hospitalizations on emotion recognition for the depression group (H3)

Statistically significant results were found for emotion recognition using the Spearman’s rank correlation coefficient ($r=$ 0.384, $p=$ 0.004) as a negative correlation was found indicating that the higher the age of onset, the fewer hits. The duration of illness, measured by the years of disease evolution, did not show a significant correlation to emotion recognition analyzed by Spearman’s rank correlation coefficient ($r= -$ 0.133, $p=$ 0.339).

Statistically significant results could not be found for emotion recognition using the Spearman’s rank correlation coefficient ($r=-$ 0.224, $p=$ 0.103). The number of hospitalizations showed to not be influential on the emotion recognition performance.

### Influence of the results of different psychometric scales on emotion recognition for the depression group (H4)

The results of some psychometric scales referring to degree of psychopathology, excessive rumination, degree of functioning and quality of life of depression group are summarized in relation to emotion recognition.

#### Psychopathology

The Spearman’s rank correlation coefficient could not find a significant influence between the results of PANAS and emotion recognition, being these results referred to Positive PANAS ($r=$ 0.075, $p=$ 0.592) and Negative-PANAS ($r=$ 0.009, $p=$ 0.949). The kind of affectivity (positive or negative) experimented on two last weeks showed to not be relevant to emotion recognition in patients.

The influence of the degree of depression measured by the BDI-II on emotion recognition was studied through two different analyses. First, Spearman’s rank correlation coefficient was carried out to measure the relationship by considering BDI-II results as a continuous variable, with non-significant influence as finding ($r= -$ 0.044, $p=$ 0.752). A second analysis was carried out by the Kruskal–Wallis test, in which BDI-II results were studied as a discontinuous variable by creating three categories, composed of minimal ($n=$ 25), mild and moderate ($n=$ 17), and severe ($n=$ 12) depression. No significant differences among groups were found ($\chi ^2_{(2)}$ = 2.573, $p=$ 0.276). BDI-II results are not a predictor of emotion recognition.

The degree of depression measured by the HRSD had no influence on emotion recognition; the Spearman’s rank correlation coefficient could not find a significant relationship between variables ($r= -$ 0.106, $p=$ 0.452). HRSD results cannot be associated with emotion recognition. The analysis of the influence between the results of STAI-T and emotion recognition did not show to be significant regarding Spearman’s rank correlation coefficient ($r= -$ 0.048, $p=$ 0.732).

#### Excessive rumination and related

Statistically significant results could not be found between depressive rumination measured by the SDRS and emotion recognition using the Spearman’s rank correlation coefficient ($r=$ 0.124, $p=$ 0.372).

The influence of IUS-12 results on emotion recognition was analyzed by considering it as continuous and discontinuous variable. As continuous variable, Spearman’s rank correlation coefficient showed no significant relationship regarding the Total IUS-12 ($r= -$ 0.133, $p=$ 0.340), the PIU ($r= -$ 0.131, $p=$ 0.344) and the IIU ($r= -$ 0.124, $p=$ 0.373). In order to convert IUS-12 performance in a discontinuous variable, the results were classified into two groups, 0 or 1, depending if they exceeded the cut-off point of the clinical reference sample^63^; the Mann–Whitney U test showed no influence referring to Total IUS-12 ($U=$ 334.0, $p=$ 0.620; cases 0, n = 25, cases 1, n = 29), PIU ($U=$ 311.0, $p=$ 0.393; cases 0, n = 24, cases 1, n=30), and IIU ($U=$ 355.5, $p=$ 0.986; cases 0, n = 23, cases 1, n = 31).

The Spearman’s rank correlation coefficient could not find a significant influence between the results of Mini-CERTS and emotion recognition, being these results referred to CET or constructive thinking ($r=$ 0.098, $p=$ 0.483), and to AAT or nonconstructive thinking ($r= -$ 0.029, $p=$ 0.834).

#### Functioning: functional assessment short test (FAST)

The FAST scale obtains outcomes that can be summarized in a value that ranges from 0 to 72, meaning 0 the best functioning of the patient and 72 the worst. No statistically significant correlation was found by the Spearman’s rank correlation coefficient between FAST and the recognition rates ($r= -$ 0.205, $p=$ 0.137). Therefore, the different values of the FAST score did not influence the number of emotions recognized.

#### Quality of life: World Health Organization Quality of Life Field Trial Version (WHOQOL-BREF)

Using the Spearman’s rank correlation coefficient, no statistical significance could be found in emotion recognition for any of the four domains that compose the scale: Physical health ($r=$ 0.190, $p=$ 0.170), Psychological ($r= -$ 0.023, $p=$ 0.871), Social relationships ($r=$ 0.118, $p=$ 0.396), and Environment ($r= -$ 0.004, $p=$ 0.975).

## Discussion

The present work has focused on evaluation of facial recognition of emotions in 54 stable patients with a diagnosis of major depression, in its comparison with 54 healthy controls matched by age, gender and educational level. To carry out the evaluation, a tool previously validated by the research team was administered, using non-immersive virtual reality with DVFs. The hypotheses raised are discussed below, in light of the results obtained.

As hypothesized in previous studies^41,42^, patients with MDD showed a lower rate of emotional recognition on virtual emotional expressions when compared to healthy controls for all emotions included, which reinforces hypothesis H1. The differences between groups were more pronounced for some negative emotions (fear, sadness and disgust). Among the depressed group, the worst recognized emotions were three of the four negative emotions presented, highlighting fear, sadness and disgust (with success rates of 48.9, 63.0% and 66.9%, respectively), while the best recognized emotions were neutral (90.3%), surprise (89.6%) and joy (84.5%). Despite the growing interest in recent years in facial recognition of emotions with DVFs, a recent review on the recognition of emotions through VR revealed that there is still not enough research on the topic that has been able to establish standardized tools, being difficult to find studies with conclusive results on all basic emotions^43^. The comparison of our results with previous similar studies is therefore controversial, as most previous studies have been conducted with behavioral instruments using static stimuli. Nevertheless, worse facial emotion recognition in depression has been found to be a robust finding, consistent with previous studies^41^. Although it is difficult to know the causes of these differences, some authors have suggested that this poorer performance may be supported by the tendency to motor slowness and indecision shown as a symptom of MDD^3^, and by a relationship between emotion recognition and alexithymia, defending that the difficulties in recognizing and categorizing emotions may be mediated by another prior impairment in the identification and naming of even one’s own emotions^44^. Regarding the recognition scores of the MDD and healthy groups in relation to age, it was observed, consistent with previous results, that the ability to recognize emotions decreases after the age of 60. In this study, the few hits were particularly relevant for the MDD group the older the participant was.

Regarding intragroup emotion recognition in depression, contrary as expected in H2, sadness did not obtain the highest rate of emotional recognition compared to the other emotions. In fact, sadness has been the second worst recognized emotion after fear. Some studies found similar results^45,46^, showing better recognition rates for mainly joy and also anger, and thus discarding the traditionally described bias for sad faces. Anyway, an important variability among studies is evidenced when this issue is reviewed^42,47^. Authors have suggested, in an attempt to explain it, that an attenuation of the positive protective bias, typical of healthy people, could be more influential than the negative bias in depressive patients when emotional recognition is required^48^, especially when ambiguous and neutral stimuli are presented^49^.

In our study, the emotions that required the longest identification time for the depression group were neutral expression and sadness. The result of neutral expression was similar in the two groups (healthy controls and patients with depression) and probably had to do with the participant waiting until the emotional transition was complete to be able to respond. The case of sadness is different. In healthy controls it was the most rapidly recognized emotion [2.16 (0.77)], while in patients with depression it was the second emotion that took the longest to recognize [5.41 (2.75)], which supports the results of previous studies.

As expected, the duration of illness and number of hospitalizations throughout life were not correlated with successful emotional recognition. Nevertheless, contrary to what was hypothesized under H3, a significant negative relationship was observed between age of onset and emotional recognition, determining the older age of onset, the less hits. In research, the age of onset on depression is usually collected as a descriptive variable of the sample, and thus, its possible influence on emotion recognition has not been studied. Future literature about this issue would be interesting in order to increase data and to be able to compare results.

Regarding hypothesis H4, the results obtained did not confirm expectations. No relationship was found between the degree of psychopathology and emotion recognition, being this finding opposed to previous findings which have found worst performance associated to the severity of depression^42,50,51^, or even attentional biases only for high levels of depressive mood^52^. The expected relationships with excessive rumination (SDRS, IUS-12, MiniCERTS), the degree of functioning (FAST) or quality of life (WHOQOL-BREF) and emotion recognition were unconfirmed as well^46^. Perhaps, the endogenous factor of depression could be something to keep in mind to interpret these unexpected results. The manual of mental disorders most used by clinicians today, in which our diagnosis are based on (DSM-5), allows clinicians to diagnose the major depressive disorder by meeting the established criteria in which several types of depression can be included. Although some specifiers are available to refine a more specific diagnosis, they are rarely considered in research. In addition, it has to be noted that all patients of the depressed sample were being medicated, which could mediate on results as suggested by previous literature^45,52–54^.

The present study has also some limitations. First, antidepressants could interfere with emotional recognition of patients with depression. In the present study, all the patients that conformed the depression group were being treated with psychotropic drugs at the time of the assessment, and therefore, it could be a limitation of this study. In the future, it would be interesting to be able to compare results of a depressed sample with and without pharmacological interventions. Neurocognitive deficits and medication effects could be reported. On the other hand, although the inclusion of the six basic emotions can be understood as a strength of our study, it could also be considered a limiting aspect because it makes it more difficult for participants to make decisions among various options. Lastly, following the line of the present study, future research in this field could be oriented to analyze the content of the wrong responses in order to investigate possible response patterns and thus identify possible emotional biases.

## Methods

### Participants

The recruitment of participants, comprising of individuals with MDD and healthy controls, was conducted over a period of 6 months (June–November 2021) at the Mental Health Service of the Complejo Hospitalario Universitario de Albacete, which serves a population of approximately 300,000 inhabitants. The authors state that all procedures contributing to this study comply with the ethical standards set by relevant national and institutional committees on human experimentation and the Helsinki Declaration of 1975, as revised in 2008. The study was granted ethical approval by the Clinical Research Ethics Committee of the hospital on April 26, 2022, with the reference number 2020/12/141.

The sample size for the study consisted of 108 participants, comprising of 54 stable patients diagnosed with MDD and 54 healthy controls from the same demographic area. Participants were deemed stable when they had achieved complete remission of depression, having been without significant symptoms for a minimum of 2 months, scoring $\le$ 6 on the Hamilton Rating Scale for Depression (HRSD), and having undergone no changes in medication in the last 3 months. The sample size was determined by the number of eligible stable patients available for inclusion in the study. A sensitivity test was performed using the G*Power program (version 3.1.9.6) to ensure that the sample size would provide an adequate level of statistical power, resulting in an effect size d = 0.640 for $\alpha$ = 0.05, power = 0.95 ($1 - \beta$), two sample groups with *n* = 54, a non-centrality parameter $\delta$ = 3.311 and a critical value *t* = 1.66. In line with our previous work^40^, participants were divided into three age groups (20–39, 40–59 and 60–79 years) and three educational levels (basic, medium and high), according to the Spanish National Institute of Statistics (INE).

Table 2 presents the sociodemographic data for both the patient and control groups. The gender and level of education were matched between the two groups, resulting in similar ages for both samples.

**Table 2:** Sociodemographic and clinical data.

	MDD group	Healthy group
Sample [n]	54	54
Gender [female:male]	34:20	34:20
Age [mean (SD)]	53.20 (13.63)	50.54 (13.72)
Age [n]		
Young (20–39)	9	10
Middle-age (40–59)	27	27
Elderly (60–79)	18	17
Education level [n]		
Basic	17	17
Medium	21	21
High	16	16
Clinical variables [mean (SD)]		
Onset age	39.80 (15.74)	
Disease evolution	12.13 (13.59)	
Hospitalizations	0.33 (0.67)	

For the group of patients diagnosed with MDD, the inclusion criteria were as follows:

- A diagnosis of depressive disorder as determined by the Structured Clinical Interview for DSM-5 (SCID).
- Clinical stability for at least 3 months prior to completing the SCID.
- Being an outpatients.
- An age between 20 and 79 years old.
- Adequate proficiency in and comprehension of the Spanish language.
- Signing an informed consent form.

The criteria for exclusion were as follows:

- Meeting diagnostic criteria for another major mental disorder on Axis I of DSM-5, with the exception of nicotine dependence.
- Presence of intellectual disability.
- Presence of a medical condition that may affect the ability to recognize facial expressions.

The inclusion criteria for healthy controls were d), e) and f) described for the depression group. The exclusion criteria were those b), c) described for the group with depression or having a personal history of mental illness.

### Data collection procedure

Each patient’s eligibility for the study was determined through a screening evaluation conducted by the referring psychiatrist during a clinical appointment. Information on sociodemographic and clinical characteristics was collected during the baseline visit, including personal and family psychiatric history, comorbid medical conditions, substance use (current and/or past), current treatment (pharmacological and psychotherapeutic), previous hospitalizations and emergency department visits.

Data collected for sociodemographic variables included patient age, gender, race, marital status, place of residence (rural/urban), education level, employment status (active, unemployed, temporary incapacity for work, pensioner, housekeeper, student), and profession. Clinical data collected included personal somatic history (including neurological), toxic personal history, psychiatric history (including diagnosis, duration of illness, number of exacerbations and hospitalizations), current treatment regimen and relevant family history. The variables and measurement instruments used in the study were as follows:

- Affectivity:
  - Spanish version^55^ of the Positive and Negative Affect Scale (PANAS)^56^. PANAS is an auto-administered scale that provides a measure of the current affectivity by rating the frequency of presenting each of 20-items (corresponding to mood states) during the last week.
- Severity of depressive symptoms:
  - Spanish version^57^ of the Beck Depression Inventory II (BDIII)^58^. It is a 21-item auto-administered scale with multiple choice answers. The obtained punctuation allows clinicians to determine the severity of depression: minimum (0–13), mild (14–19), moderate (20–28) and severe depression (29–63).
  - Spanish version^59^ of the 17-item Hamilton Rating Scale of Depression (HRSD)^60^. This scale was designed for patients previously diagnosed with depression in order to quantitatively assess the severity of symptoms and assess patient changes. It is composed of 17 items, which in turns have between three and five possible answers, scored from 0 to 2 or 0 to 4, respectively.
- Anxiety trait:
  - Spanish version^61^ of the Trait subscale of State-Trait Anxiety Inventory (STAIA/STAI-T)^62^, which assesses self-reported anxious symptoms, referring to general or chronic experience of anxiety. We used the 7 items with a Likert-response format (0–3) of the STAIT^63^, since these items were identified through a factor analysis to be more strongly correlated with trait anxiety than depression^64^.
- Rumination and related:
  - Spanish version^65^ of the Short Depressive Rumination Scale (SDRS) from the Leuven Adaptation of the Rumination on Sadness Scale (LARRS)^66^. It is an auto-administered scale formed by 4 items about rumination trends when depressed, in which frequency is rated from 1 to 10.
  - Spanish adaptation of the Intolerance of Uncertainty Scale, IUS-12^67^. This is a short scale of 12-item that assesses the level of intolerance of uncertainty using a Likert-response format (1–5). IUS-12 allows clinicians not only to evaluate the intolerance of uncertainty, but also to evaluate two more constructs: prospective anxiety (PIU) and inhibitory anxiety (IIU).
  - Spanish adaptation of the Mini-Cambridge-Exeter Repetitive Thinking Scale (Mini-CERTS)^68^, a 15-item scale with a Likert-response format (1–4) that classifies rumination into two dimensions: Concrete and Experiential Thinking (CET) or constructive thinking, and Analytic and Abstract Thinking (AAT) or nonconstructive thinking.
- Functionality and quality of life:
  - The Functioning Assessment Short Test, FAST^69^. FAST is a 24-item scale designed to assess an individual’s functioning in six specific areas: autonomy, occupational functioning, cognitive functioning, financial issues, interpersonal relationships, and leisure time. The scale is quick and easy to administer, and responses are recorded using a Likert-response scale ranging from 0 to 3, with 0 indicating no difficulty and 3 indicating severe difficulty.
  - Spanish version^70^ of the WHOQOL-BREF Brief World Health Organization Quality Of Life, WHOQOL-BREF^71^. It is composed of 26 items: 24 are referred to the 24 aspects of the original WHOQOL-100, and the remaining 2 are global items about quality of life and general health. The answer format is a Likert-response scale (1–5), which correction gives rise to the evaluation of 4 domains: Physical, Psychological, Social relationships and Environment health.

The recruitment of healthy controls was performed in the same demographic area as the patients and primarily from similar cultural and social groups. The research team designed a data collection notebook that included both sociodemographic and clinical information. The sociodemographic data collected were identical to those collected from the patients with depression. In addition, clinical data such as personal somatic history (including neurological history), personal toxic and psychiatric history, and relevant family history were also collected. The data collection was conducted in a single individual session that lasted 30 min. If the participant met the study’s inclusion criteria, they were then presented with the facial stimulus.

All participants gave informed consent after receiving a detailed explanation of the study and before participating in the experiment. The acquired data were stored anonymously using dissociated databases.

### Experimental procedure

**Figure 1:**
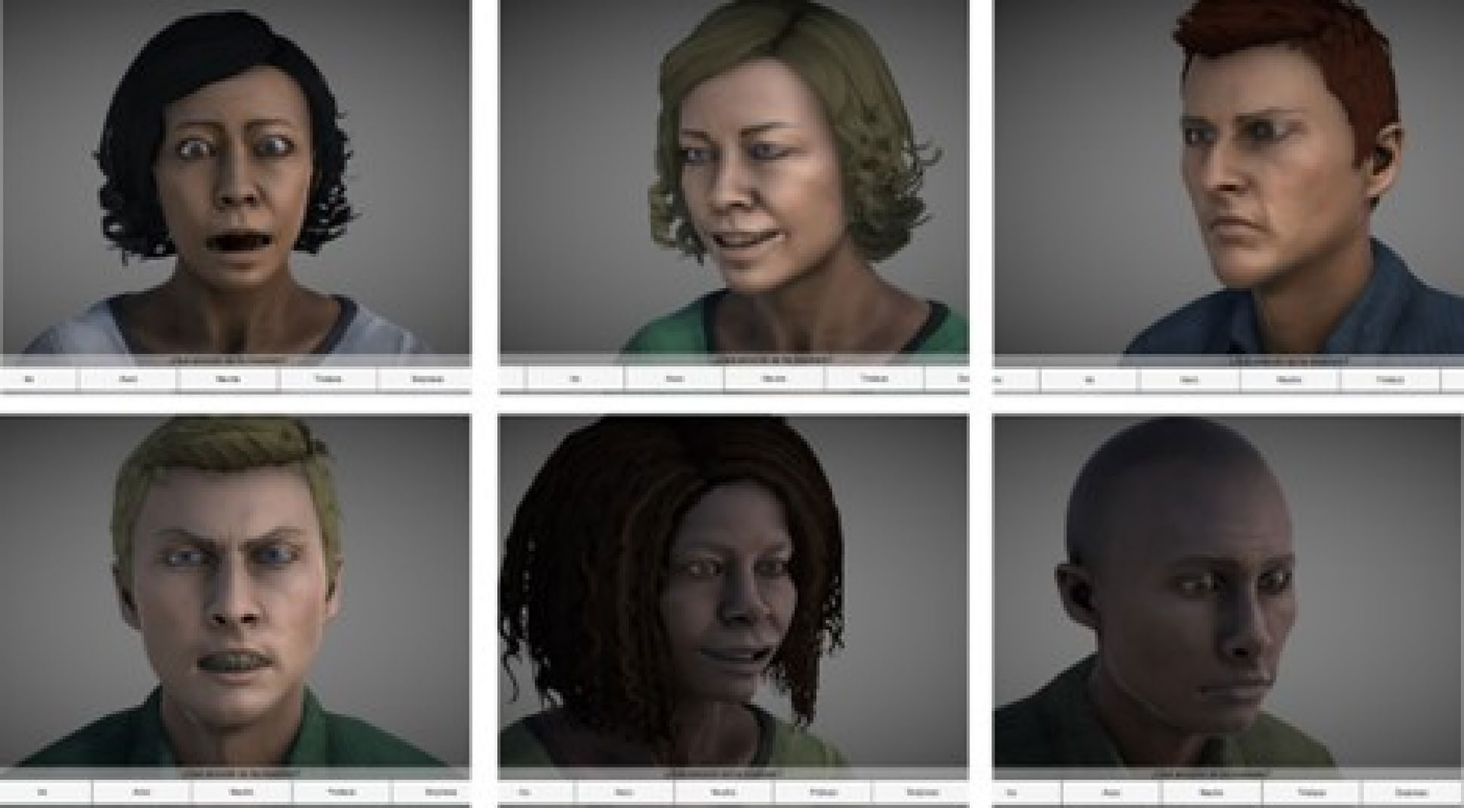
A few DVF samples created by the authors using Unity version 2020.2.2f1 (https://unity.com/).

The experiment was conducted in a single session, lasting between 40–50 min and including the administration of both sociodemographic and clinical scales. To begin, participants were given a brief tutorial to familiarize themselves with the task at hand. Throughout the session, all participants were presented with 52 DVFs on a 27-in. computer screen (see samples in Fig. 1). Each DVF began with a neutral expression, then transitioned to one of the six basic emotions (happiness, sadness, anger, fear, disgust, and surprise) or remained neutral before ending on a neutral expression.

The total presentation time for each DVF was 2 s. After viewing each DVF, participants were required to identify the basic emotion depicted by selecting one of the seven options provided on the screen below the DVF. Of the 52 faces presented, 50% were interspersed with less dynamic movement (only the most characteristic facial features of each emotion moved) and 50% featured more dynamic faces (movement was represented by facial features and movements of the neck and shoulders).

The set of DVFs employed in the study were presented from three different perspectives: 50% were displayed in a frontal view, 25% were presented from the right profile, and 25% were presented from the left profile. Additionally, other physical characteristics were also taken into consideration in the selection of the avatars used. Specifically, the DVFs included two white avatars, both approximately 30 years of age, with varying eye color, skin tone, and hair characteristics, as well as two black avatars and two avatars of older age. Out of the 52 DVF presented, 8 were of black race and 8 were of older age. The complete characterization of the DVFs used in the study can be found in our previous experiment, in which they were validated by a sample of 204 healthy individuals^26^.

### Statistical analysis

The statistical analyses were conducted utilizing IBM SPSS Statistics (version 24) and Microsoft Excel. Descriptive statistics were employed to analyze quantitative variables, including the mean and standard deviation, and qualitative variables were represented by percentages. As the distribution of hits did not follow a normal pattern, non-parametric tests were primarily utilized to test the hypotheses, with statistical significance set at a *p* value< 0.05. Comparisons between groups were performed using the Mann Whitney U test when comparing only two groups, and the Kruskal–Wallis test for three or more groups. The correlation between variables was examined utilizing the Spearman’s rank correlation coefficient.

## Data Availability

The datasets generated during and/or analyzed during the current study are available from the corresponding author on reasonable request.
